# A Case Report of Xanthelasma and the Associated Differential Diagnosis

**DOI:** 10.7759/cureus.81971

**Published:** 2025-04-09

**Authors:** Braden Van Alfen, Jonny Hatch, Mark Conley, Sathishkumar Seeliyur Duraiswamy

**Affiliations:** 1 Texas College of Osteopathic Medicine, University of North Texas Health Science Center, Fort Worth, USA; 2 Dermatology Residency Program, Trinity Health Ann Arbor Hospital, Ypsilanti, USA; 3 Hospital Medicine, Baylor All Saints Medical Center, Fort Worth, USA

**Keywords:** erdheim–chester disease, lipoid proteinosis (lp), necrobiotic xanthogranuloma, ocular sarcoidosis, primary biliary cholangitis (pbc), xanthelasma

## Abstract

Xanthelasma, also known as xanthelasma palpebrarum, is a harmless, soft yellow plaque on or near the eyelids. A significant proportion of patients with xanthelasma have an underlying disease causing hyperlipidemia and should be evaluated for underlying causes.

A 61-year-old woman presented to the emergency room for abdominal pain and lower extremity edema for two weeks. On physical exam, the patient was jaundiced with scleral icterus and asymptomatic bilateral yellow plaques on the medial aspect of the upper eyelids. The patient had a history of primary biliary cholangitis (PBC), and previous lab results were positive for anti-mitochondrial antibodies and Anti-smooth muscle antibodies. The patient’s history and presentation were sufficient for the diagnosis of xanthelasma.

This case report describes an interesting case of xanthelasma and details the differential diagnosis that should be considered. The differential diagnosis includes necrobiotic xanthogranuloma, Erdheim-Chester disease, lipoid proteinosis, palpebral sarcoidosis, syringoma, sebaceous hyperplasia, and nodular basal cell carcinoma. Although xanthelasma can be easily diagnosed in some cases, other conditions on the differential should be considered to avoid delay in patient care.

## Introduction

Xanthelasma, also known as xanthelasma palpebrarum, is a harmless yellow plaque on or near the eyelids and may be soft, chalky, or semi-solid [1]. Approximately half of patients with xanthelasma have underlying conditions causing hyperlipidemia and should be evaluated for underlying causes [2]. Primary biliary cholangitis (PBC) is one known cause of xanthelasma and is an autoimmune condition that contributes to the destruction of bile ducts, leading to cholestasis. Affected individuals may be asymptomatic, presenting with abnormal liver chemistry or may present with jaundice, pruritus, and fatigue [3]. Additionally, the bile duct destruction may lead to cirrhosis and portal hypertension.

## Case presentation

**Figure 1:**
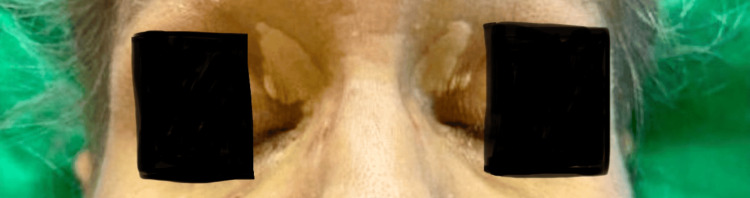
Bilateral yellow plaques on the medial eyelids consistent with xanthelasma

A 61-year-old female with a history of psoriatic arthritis, hypothyroidism, and liver cirrhosis presented to the emergency room for abdominal pain and lower extremity edema for two weeks. On exam, the patient was jaundiced with scleral icterus. Her abdomen was soft and distended. Her bilateral lower extremities were erythematous with 2+ pitting edema. She presented with asymptomatic bilateral yellow plaques on the medial aspect of the upper eyelids (Figure 1). 

The patient had a history of PBC. The patient had had a previous workup for liver disease that included a liver biopsy demonstrating a predominantly lymphocytic portal infiltrate and few plasma cells, neutrophils, or eosinophils. Additionally, a classic histological hallmark of PBC was observed: florid duct lesions, or destruction of interlobular bile ducts by poorly formed portal epithelioid granulomas. Labs were positive for anti-mitochondrial antibodies and anti-smooth muscle antibodies, correlating with a diagnosis of PBC. Later lab evaluation revealed high antinuclear antibody titers at 1:640 and anti-mitochondrial antibody titers at 1:1280. In addition, she had palmar xanthomas, which are highly correlated with PBC [4]. No further biopsy was taken as the patient’s history and exam were sufficient for diagnosing xanthelasma.

## Discussion

Xanthelasma plaques are generally not itchy or painful and were not bothering the patient in the case presented. Xanthelasma are most common in patients of Mediterranean or Asian ancestry [5], and only half of patients with xanthelasma have high cholesterol levels [1]. Some studies suggest that even with normal cholesterol levels, xanthelasma is a risk factor for cardiovascular disease [6]. Lesions are typically distributed symmetrically on the medial portion of the upper eyelids, as was seen in this patient [6]. 

Patients with xanthelasma may warrant a full liver panel, thyroid function test, and fasting blood glucose [2]. Our patient had a history of PBC, an autoimmune disorder leading to gradual destruction of intrahepatic bile ducts [3]. Bile duct destruction leads to cholestasis and may subsequently lead to cirrhosis and portal hypertension. The cholestasis leads to abnormal lipid profiles that can subsequently result in xanthelasmas [3]. As PBC progresses to cirrhosis, ascites, spider nevi, and edema may be noted on exam [3]. This progression accounts for the history of cirrhosis and bilateral lower extremity edema in our patient. Most common in middle-aged women, PBC is thought to occur due to an interaction between environmental triggers and a genetic predisposition [3]. Human leukocyte antigens (HLA) associated with PBC include DRB1, DR3, DPB1, DQA1, and DQB1, and environmental triggers include cigarette smoke, nail polish, and various xenobiotics [3]. The anti-mitochondrial antibody is the most specific antibody associated with PBC and is found in 85% of cases [3]. Around 40% of PBC patients have skin complaints, including xanthomas (a broader term for cholesterol buildup underneath the skin) and pruritus. Approximately 50% of PBC patients have elevated lipid profiles manifesting as xanthomas or xanthelasmas [3]. 

Elevated triglyceride levels may be present in dermatologic conditions other than xanthelasma, including eruptive xanthomas and psoriasis, and may be caused by dermatological therapies such as vitamin A derivatives [7]. Patients should be advised to implement lifestyle modifications to lower cholesterol and triglycerides, but xanthelasmas do not typically resolve or regress with lifestyle modifications [2]. Xanthelasma does not have premalignant potential. For ambiguous cases, excision and histopathological examination should be performed [6]. Other treatments include cryotherapy, laser treatment or chemical peels with trichloroacetic acid or radiofrequency [6]. Laser treatments are often the preferred method, and recurrence is common regardless of the removal option [2]. 

Histopathology of xanthelasma would have shown a perivascular inflammatory infiltrate with mononucleated and multinucleated foamy histiocytes characterized by intracellular fat deposits [2]. These xanthoma cells are found in the upper reticular dermis [6]. The papillary dermis, epidermis, and subcutaneous layer generally do not exhibit the intrahistiocytic vacuoles containing cholesterol that are found in the reticular dermis [6]. 

Differential diagnosis includes necrobiotic xanthogranuloma, Erdheim-Chester disease, lipoid proteinosis, palpebral sarcoidosis, syringoma, sebaceous hyperplasia, and nodular basal cell carcinoma. Necrobiotic xanthogranuloma (NXG) is a rare, chronic granulomatous disease presenting with yellowish or violaceous plaques and nodules on the periorbital skin [8]. Up to 80% of patients with NXG have monoclonal gammopathy [8]. In addition, patients with NXG have an increased likelihood of plasma cell dyscrasias, including myeloma and should be screened appropriately. NXG presents in the periorbital skin rather than the eyelids, is rarer, and has a far less understood etiology as compared to xanthelasma palpebrarum. 

Erdheim-Chester disease is a multisystem non-Langerhans cell histiocytosis with fibrosis characterized by CD68+, CD1a-, and S100- expression. ECD commonly presents with symmetrical osteosclerosis of long bones, diabetes insipidus, exophthalmos, and xanthelasma-like lesions [9]. Dermatologic manifestations of ECD occur in just 33% of patients and most commonly present as xanthelasma-like lesions [9]. The xanthelasma-like lesions seen in ECD are far rarer than the xanthelasma caused by PBC. PBC does not have skeletal manifestations, diabetes insipidus or major effects on the eyes. 

Lipoid proteinosis is another rare condition with only 400 reported cases in the literature [10]. This is an autosomal recessive condition with hyaline-like deposits in the skin and other tissues and organs, and occurs due to a loss-of-function mutation in extracellular matrix protein 1 (ECM1) that encodes glycoproteins necessary for the basement membrane and ECM structure integrity and skin adhesions [10]. Histologically, it presents with Periodic acid-Schiff positive deposits within the papillary dermis and at the dermal-epidermal junction [10]. This condition typically presents in a patient with hoarseness due to involvement of vocal cords and cutaneous manifestations include vesicles or bullae and hemorrhagic crusts on sites of trauma, typically on the face [10]. Lipoid proteinosis lesions tend to appear as a string of nodules along the lid margin and have mucocutaneous involvement that is not typically associated with xanthelasma [6].

Ocular sarcoidosis may involve any part of the eye, including the eyelid (palpebral sarcoidosis) [11]. Sarcoidosis presents as erythema of the eyelids and may have mass lesions varying from papules to a large mass [11]. Histology shows noncaseating granulomas of the eyelid, just as sarcoidosis would demonstrate in other organs. While masses may be present, periorbital erythema and swelling may be the only cutaneous manifestations of ocular sarcoid [11].

While xanthelasma is far more prevalent than the other diagnoses on the differential, providers should be aware of these conditions. Xanthelasma classically presents with yellow lesions bilaterally above the medial canthus [2]. Deviation from this classic presentation should raise suspicion for other possible causes that resemble xanthelasma. The diagnosis of xanthelasma is typically clinical and does not require biopsy; however, if there is uncertainty of the diagnosis or suspicion of these rare conditions, biopsy is warranted [2].

## Conclusions

This case presents the clinical manifestations of xanthelasma associated with PBC. Xanthelasma is a benign yellow plaque that often appears on the bilateral medial eyelids and is often associated with an underlying disease process. While this patient presented with a history and previous workup characteristic of PBC, cases resembling xanthelasma without the characteristic features of PBC may need a more in-depth workup to rule out rarer causes of skin manifestations that resemble xanthelasma. Physicians and other care providers should be aware of these rare conditions to avoid delays in patient diagnosis and treatment. 
